# Predictors of DMPA-SC continuation among urban Nigerian women: the influence of counseling quality and side effects^☆^^☆☆^

**DOI:** 10.1016/j.contraception.2018.04.015

**Published:** 2018-11

**Authors:** Jenny Liu, Jennifer Shen, Nadia Diamond-Smith

**Affiliations:** a Institute for Health and Aging, Department of Social and Behavioral Sciences, School of Nursing, University of California, San Francisco, 3333 California Street, Suite 340, San Francisco, CA 94118, USA; b Institute for Health Policy Studies, School of Medicine, University of California, San Francisco, 3333 California Street, Suite 266D, San Francisco, CA 94101, USA; c Global Health Sciences, Department of Epidemiology and Biostatistics, School of Medicine, University of California, San Francisco, 550 16th Street Mission Hall, San Francisco, CA 94158, USA

**Keywords:** Depot medroxyprogesterone acetate (DMPA), Subcutaneous injection, Contraception, User experience, Side effects, Continuation

## Abstract

**Objectives:**

In 2015, private healthcare providers in Nigeria introduced DMPA-SC (depot medroxyprogesterone acetate administered subcutaneously) into the method mix. We aimed to [1] examine the sociodemographic predictors of continued DMPA-SC use after 3 months, and [2] characterize the additional influences of contraceptive counseling quality and experiences of side effects on continuation.

**Study design:**

From March to August, 2016, we conducted phone interviews with a convenience sample of women obtaining DMPA-SC from selected providers to survey them about their experience obtaining an initial dose of DMPA-SC. Study coordinators contacted women again about 3 months later after when they were due for reinjection. We used logistic regressions to examine the likelihood of having obtained a subsequent dose of DMPA-SC at follow-up as predicted by sociodemographic characteristics, a quality of counseling indicator based on responses to a 14-item scale, and reports of side effects experienced.

**Results:**

Of the 541 DMPA-SC users who completed the first survey, 311 were reached again via phone after 3 months to conduct a second survey. Multivariate results for sociodemographic predictors of continued DMPA-SC use show that those with some college education or more (OR=2.79; 95% CI: 1.09–7.14), and those with four or more children (OR=2.89; 95% CI: 1.09 0 7.67) were more likely to obtain another dose. Our summary quality measure showed that women overall rated the quality of their initial counseling session high. Logistic regressions indicated that higher quality during the initial counseling session is related to the likelihood of getting another dose of DMPA-SC (OR=2.04; 95% CI: 1.12–3.47) whereas experiencing more bleeding reduced the likelihood of continuation after 3 months (OR=0.15; 95% CI: 0.07–0.34).

**Conclusions:**

Among urban Nigerian women, both counseling quality and experiencing side effects were important factors in predicting continued use of DMPA-SC after 3 months. These findings are consistent with previous studies of DMPA and injectable contraception continuation.

**Implications:**

New contraceptive methods that are designed for increased access and ease of use, combined with high quality provision, have potential to increase contraceptive use in settings with low levels of contraceptive prevalence. Higher quality counseling can help encourage women's continuation of a new injectable contraceptive method at 3 months.

## 1 Introduction

Effective family planning requires contraceptive adherence. However, due to a variety of factors—cost, convenience, method dissatisfaction, experiences of side effects, partner disapproval, and limited method options—many women discontinue contraception, which contributes to high rates of unwanted fertility and overall fertility [1], [2], [3], [4]. Researchers and family planning practitioners alike believe quality of contraceptive counseling and care is critically important for method continuation [5]. High quality providers may be better equipped to give contraceptive counseling, and may be more effective at informing women of potential side effects, thereby increasing the likelihood that women manage or tolerate these experiences and continue with their method.

Although many studies have explored the relationship between quality of care and rates of discontinuation, the empirical evidence supporting this hypothesis remains inconclusive. A study across 15 countries showed that between 7 and 27% of women discontinued contraception because of poor service quality [6]. Improvements in the quality of care at health centers, either engendered through policy changes, as seen in Senegal [7], or through health worker training programs incorporating special emphases on contraceptive counseling, such as in Kenya [8], have led to increased contraceptive continuation among women. Such improvements may have influenced clients' perceptions of quality of care, which is associated with continued contraceptive use, as found in Bangladesh [9]. However, in Peru, no significant differences in continuation rates were found between a cohort that received family planning counseling from providers trained on a jobs-aid assisted counseling strategy and a control cohort [10]. Similarly, in the Philippines, a longitudinal family planning provider program was implemented but did not lead to any significant effects on family planning outcomes [11].

Furthermore, each contraceptive method has unique features which also influence the likelihood of method and contraceptive continuation. Ali et al. report method-specific discontinuation probabilities across 19 Demographic and Health Survey (DHS) countries, and find 12-month discontinuations rates of 50.4% for condoms, 43.5% for the pill, 40.6% for injectables, and 13.1% for IUDs amid an overall rate of 37.7% [3]. In both developed and developing country contexts, IUDs have the highest continuation rates compared to other methods, possibly due to higher costs of removal associated with the method [12], [13], [14], [15].

Since 2014, a new, easy-to-administer, all-in-one, 3-month DMPA-subcutaneous (DMPA-SC; i.e., Sayana Press**®** formulation for depot medroxyprogesterone acetate) contraceptive injectable has been introduced in more than 14 countries, including Nigeria. DMPA-SC is a low-dose formulation of DMPA (104-mg/0.65 mL), with slower rate of absorption and equivalent efficacy, as well as high patient satisfaction [16]. Many hypothesized that DMPA-SC would be a ‘game changer’ [17] for increasing access to contraception in low resource settings because of its potential for home- and self-injection and ease of use [18], with cohort studies in Senegal showing the feasibility of self-injection [18]. Recent studies show that discontinuation rates of self-administered DMPA-SC were not different and even reduced compared to provider-administered DMPA-IM (i.e., DMPA-intramuscular; e.g., Depo Provera) [19], [20], [21]. Yet, little is known about other predictors of adherence to DMPA-SC, including quality of care and experiences of side effects.

Compared to IUDs and other long-term methods, continuation of injectable contraceptives is more difficult to maintain. Even as injectable contraceptives are gaining popularity in low-resource settings [22], [23], [24], especially for young women [25], continuation on injectable contraception is low in many settings [26], [27]; discontinuation typically occurs within one year with estimated rates of up to 77%, and particularly high rates of drop-off observed after the first and second injections [3], [26], [28], [29]. In one study in Nigeria, more than 50% of sampled women using DMPA-IM did not continue within one year [30]. The most frequently cited reasons for discontinuation were incompatibility with using a specific method, side effects, and desire for pregnancy [3], [27]. For DMPA-SC, emerging evidence shows that reasons for discontinuation include late reinjection, access challenges/stockouts, partner disapproval, irregular bleeding and weight changes [20], [31]. However, from studies in the U.S., China, Mexico, and South Africa, women who received more intensive structured counseling, including discussion about side effects, were more likely to continue DMPA use [26], [32], [33], [34], suggesting that the inclusion of side effects within contraceptive counseling can be one way to encourage method continuation.

In the current study, we use data collected during the introduction of DMPA-SC in Nigeria to examine DMPA-SC users' decision to continue on the method. DMPA-SC was introduced into the market in Nigeria in 2015 as part of larger efforts to increase method offerings and overall access to contraception. Although modern contraceptive use among all women in Nigeria is extremely low (11% in 2013), after condoms, injectables are the most popular method among current users, accounting for 23% of modern methods used. [35]. For the purposes of monitoring and evaluation of the DMPA-SC introductory program in Nigeria, we collected data on DMPA-SC users obtaining the product from selected private sector providers in seven South West states where the program was first introduced. Private providers included for-profit clinics, maternity homes, pharmacies, patent and proprietary medicine vendors, and individually practicing clinicians (e.g., physicians, nurses/midwives). The overall findings and lessons learned from this effort, including detailed accounting of data collection methods, are reported elsewhere [36].

In this study, we examined users' responses to two phone surveys conducted 3–5 months apart to [1] assess the sociodemographic predictors of continued DMPA-SC use at 3 months, and [2] characterize the additional influences of contraceptive counseling quality and experiences of side effects on continued DMPA-SC use. Although our convenience sample of women is primarily from urban areas, this is the first study to empirically assess whether socioeconomic and demographic backgrounds, as well as quality of family planning counseling, are predictors of DMPA-SC continuation among consumers at large obtaining the product from an array of private sector providers that dominate the provision of contraceptive products in Nigeria [31]. Since this study was not conducted in a controlled setting, our results may help inform how future scaled DMPA-SC programs may better serve their broad base of clients under real world conditions.

## 2 Materials and methods

### 2.1 Data collection

Between March and June 2016, we recruited healthcare providers across seven South West states (Ekiti, Kwara, Lagos, Ogun, Ondo, Osun, Oyo). The product's distributor, a contraceptive social marketing firm primarily supplying private sector providers, generated a list of 358 providers that had purchased DMPA-SC, including hospitals/clinics, retail drug outlets, and licensed Community Health Extension Workers (CHEWs) hired and trained to conduct proactive community-based distribution. After an initial cleaning of the provider list and eliminating providers with missing or incorrect data, we attempted to contact 316 providers for participation. Provider participation was entirely voluntary, and providers who consented were offered 1000 Naira (~US$5.00) in mobile phone credits in return for participation. In total, 205 providers consented to participate, of which 50% were from Lagos and 21% from Oyo; by profession, over 40% were nurses or midwives, and about 30% were CHEWs. Participating providers were asked to assist in the recruitment of DMPA-SC users by recording the names and phone numbers of customers who purchased an injectable contraceptive (of any type) and identifying customers who agreed to be contacted for a phone survey.

**Fig. 1:**
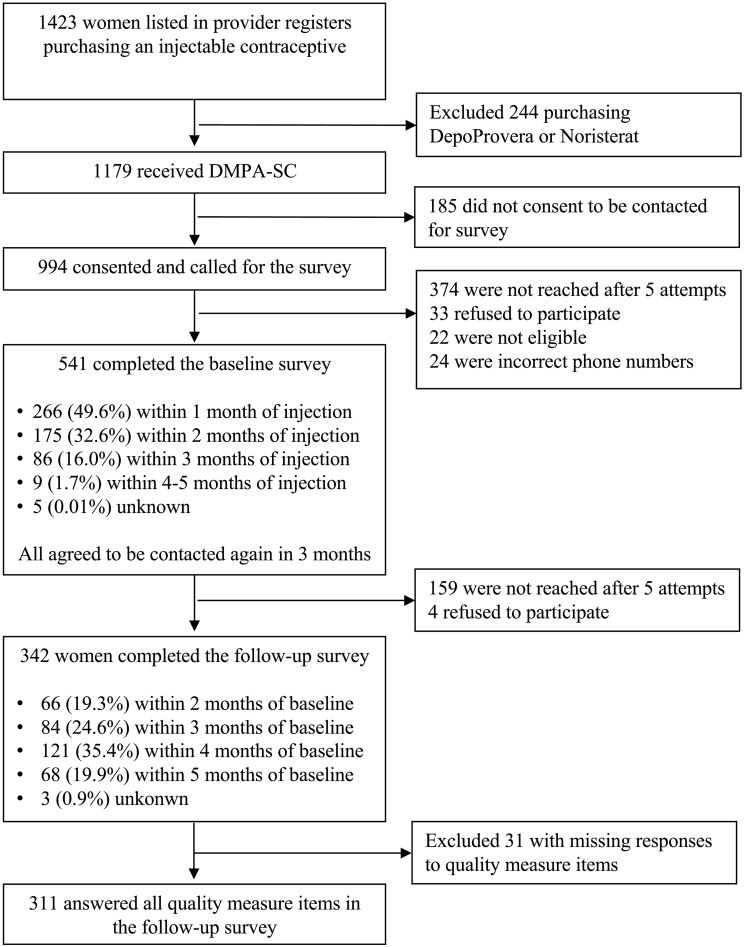
Participant recruitment, survey, and interview. Of 1423 women listed in providers' registers, 1179 purchased DMPA-SC, and all DMPA-SC customers who consented to be contacted for a survey (N=994) were called to complete an initial survey. Of 541 women completing the initial phone survey about their recent experience obtaining a dose of DMPA-SC, all verbally consented to be contacted again to complete a second phone survey about care-seeking for a subsequent dose of DMPA-SC. Of the 342 women completing a second follow-up phone survey, the 311 women who answered all quality measure items were included in this analysis.

Of 1423 women listed in providers' registers, 1179 purchased DMPA-SC. We called all DMPA-SC customers who consented to be contacted (N=994) to complete an initial survey administered by a trained, bilingual (in Yoruba, the dominant local language) interviewer over the phone, lasting 15–20 min, and compensated with 200 Naira (~US$0.57) of mobile phone credits. Of the 944 women called, 541 women completed the initial phone survey about their recent experience obtaining a dose of DMPA-SC; 374 women were not able to be reached after up to five attempts, 33 refused to participate, 22 were not eligible, and 24 phone numbers were incorrect. About half (N=266; 49.6%) were contacted within one month of their injection, 32.6% (N=175) were contacted within 2 months, 16.0% (N=86) within 3 months, and 1.7% (N=9) within 4–5 months.^1^ About 3 months later, timed for after respondents were due for a reinjection (range 2.2–5.6 months; median 4.7 months), all 541 respondents who verbally consented to be contacted again during the initial phone survey were called to complete a second phone survey about care-seeking for a subsequent dose of DMPA-SC. During the follow-up call, women were again verbally consented to participate before conducting the survey (about 5–10 min) and compensated with 100 Naira (~US$0.29) of mobile phone credits. Of the 342 women completing a second follow-up phone survey, the 311 women who answered all quality measure items were included in this analysis. Fig. 1 displays the recruitment and longitudinal follow-up of women in the study.

### 2.2 Quality measures

The first survey round included questions on three different aspects of quality (see Table 1): information given, interpersonal communications, and choice of method. These quality measures were based on the domains outlined in the Bruce-Jain framework, and informed by items used by subsequent researchers measuring various domains of the quality of family planning services [37], [38]. After collating quality measures employed by different contraception studies [5], [39], [40], we selected 16 items that covered the three quality domains in this study. These questions were pre-tested in February 2016 with women who had been administered DMPA-SC during outreach events held by the product's supplier and who verbally consented to be contacted for a phone survey. During the pre-test, we aimed to assess women's comprehension over the phone, completion time, and response fatigue. Primarily due to reasons of poor phone connection quality, which compromised user comprehension, and respondent fatigue, we chose to restrict response options to binary choices (i.e., “yes” or “no”) to the extent possible; categorical responses were retained for two items (see below).

**Table 1:** Quality items and summary measures (N=311).

	Positive responses	
Items	n	%
Information given		
Provider asked if you had ever used contraception before	283	91.1%
Provider asked if you had ever experienced any side effects from another contraceptive	271	87.1%
Provider asked you wanted to have more children in the future	278	89.4%
Provider asked if you had any health issues (e.g., infections, high blood pressure)	237	76.2%
Provider described possible side effects of DMPA-SC	230	74.0%
Provider told you what to do if you had problems with DMPA-SC	231	74.3%
Provider told you how long it protects against pregnancy	310	99.7%
Provider asked if you were pregnant/do anything to find out if you were pregnant	284	91.4%
Interpersonal		
Felt comfortable to ask questions	310	99.7%
Have enough privacy during the visit	300	96.5%
Felt that the waiting time was reasonable	276	88.7%
Choice		
Provider had no or slight preference for which method you should use (vs. moderate/strong preference)	10	3.2%
Felt that the information given to you was just right (vs. too little/too much)	304	97.7%
Provider mentioned methods other than DMPA-SC	141	45.3%
Summary measures		
Summary score (range: 0–14) mean [SD]		11.1 [1.76]
High quality indicator (score ≥ median 12)	176	56.6%

To construct summary quality measures for analysis, we excluded two items that were asked in the survey under interpersonal communications (i.e. “Did you feel the provider treated you respectfully?” and “Do you believe that the information that you shared about yourself with the provider will be kept confidential?”) because all women responded positively, thus yielding no variation for analysis. For all quality items, we constructed a binary indicator for the participant's affirmative response to the positive quality outcome, which was then summed across all items to create a composite continuous summary measure (range 0–14). For non-binary quality items of providers' preference for the choice of method (none, slight, moderate, or strong) and information given (too little, just right, too much), we categorized the responses into a binary variable. Based on findings from previous studies of patient preferences for shared and informed decision-making, providers perceived to have no or slight preference for the choice of method were considered to be indicative of higher quality than those perceived to have had a moderate or strong preference [41]. Similarly, the level of information that was perceived to be “just right” was considered to be indicative of higher quality than either “too much” or “too little.”

**Fig. 2:**
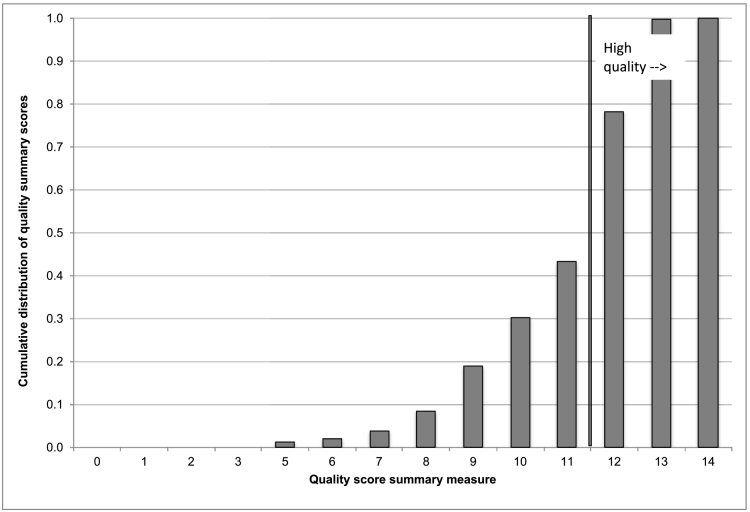
Cumulative distribution of the summary quality measure. Dummy variables indicating the higher quality outcome for all 14 item measures were summed to create a continuous quality index (range 0–14). Based on the resulting distribution of the summary index, we then created a dummy variable indicator for “high quality.” As has been done in other studies involving quality of care indices [42], we use the median quality score as the cutoff for “higher quality” versus “low quality. In our case, “high quality” is defined as a summary score greater than or equal to the median value of 12, the threshold at which a large jump is also observed in the cumulative distribution of the quality summary score. Quality summary scores of 12 or above comprised 44.7% of the sample.

Based on the resulting distribution of the summary measure, we then created a dummy variable indicator for “high quality.” As has been done in other studies involving quality of care indices [42], we use the median quality score as the cutoff for “higher quality” versus “low quality. In our case, “high quality” is defined as a summary score greater than or equal to the median value of 12, the threshold at which a large jump is also observed in the cumulative distribution of the quality summary score (see Fig. 2). Quality summary scores of 12 or above comprised 44.7% of the sample.

### 2.3 Side effects

In the follow-up survey round, all respondents were asked if they had experienced side effects associated with the injection. Respondents could report multiple side effects if relevant. Among the 304 women who responded to this question, we separated these responses into four categories to include as predictors in the multivariate regression: no side effects experience, less/no bleeding, more bleeding, and all others (which included responses such as headaches, nausea, or dizziness, weight gain or loss, fatigue, fever, malaria, upset stomach, and unspecified general body reactions).

### 2.4 Outcome: Continuation of DMPA-SC

In the follow-up survey round, we asked women if they had obtained another dose of DMPA-SC since the time of the first phone survey. We constructed a dummy variable to indicate self-reported continued use for those who reported obtaining another dose; all others who did not obtain another dose since the first phone survey, including those who stated their intent to do so, were categorized as not continuing DMPA-SC use.

### 2.5 Data analysis

We conducted multivariate analyses using logistic regression to predict DMPA-SC continuation with our quality measures and individual background characteristics (i.e. age, marital status, education, religion, parity, wealth quintile,^2^ state of residence, type of provider where the initial dose of DMPA-SC was purchased, and whether the woman had previously used some form of modern contraception prior to initiating DMPA-SC). Standard errors were corrected for heteroskedasticity and clustered by provider to account for autocorrelation among women attending the same provider.

### 2.6 Ethics statement

The Institutional Review Board at the University of California, San Francisco (IRB# 15–18,353) and the Nigeria Health Research Ethics Committee (NHREC/01/01/2007–06/01/2016) approved this research.

## 3 Results

### 3.1 Sample characteristics

Our convenience sample of DMPA-SC users (N=311; Table 1) primarily comprised of married (97.1%) women aged 25 or older (55.6% 25–34; 37.0% 35+) with secondary (58.2%) or college/university (28.3%) schooling and at least two children (86.2%). Nearly 70% were in the wealthiest quintile, and 66% obtained their initial DMPA-SC dose from a CHEW conducting proactive community-based distribution. Approximately 75% of women were from three states—Lagos, Ogun, and Oyo—which encompassed the main urban centers in the region. Our analysis of attrition found that women who were lost to follow-up were significantly more likely to be younger, unmarried, and have fewer children (see Supplementary Table S1). Of those included in the follow-up survey, 49.7% of women had obtained another dose of DMPA-SC.

### 3.2 Quality measure

Table 1 summarizes the items included our quality measure. Positive responses across quality items were nearly all high with two exceptions in the domain of method choice. Only 3.7% reported that the provider had no or little preference for which method she should choose. In addition, 45.3% reported that the provider mentioned a contraceptive method other than DMPA-SC. Our analysis of attrition also found that responses for women who were lost to follow-up were not significantly different from those women completing the follow-up survey for each quality item (see Supplementary Table S2). When summing across all items, the mean score was 11.1 with 56.6% registering a score greater than or equal to the median of 12. The Cronbach's alpha for the summary score was 0.63.

### 3.3 Side effects

**Fig. 3:**
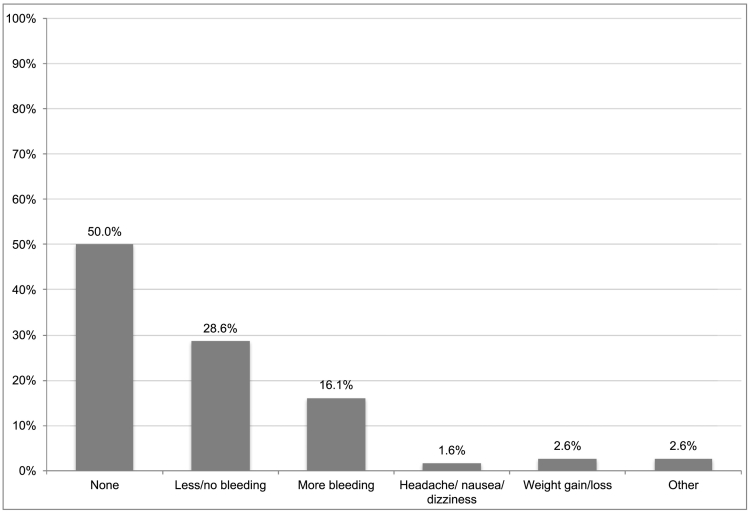
Side effects experienced. About half of respondents reported not experiencing any side effect (50.0%) while 28.6% indicated that they had less or no bleeding and 16.1% reported more bleeding. A small number of women reported headaches, nausea, or dizziness (1.6%), changes in weight (2.6%), and a variety of other side effects (2.6%).

Fig. 3 shows the side effects that women reported experiencing. About half of respondents reported not experiencing any side effect (50.0%) while 28.6% indicated that they had less or no bleeding and 16.1% reported more bleeding. A small number of women reported headaches, nausea, or dizziness (1.6%), changes in weight (2.6%), and a variety of other side effects (2.6%).

### 3.4 Regression results

Multivariate results for sociodemographic predictors of continued DMPA-SC use (Table 2) showed that those with some college education or more (OR=2.794; 95% CI: 1.093–7.140), and those with four or more children (OR=2.888; 95% CI: 1.087–7.668) were more likely than their less educated and lower parity counterparts to obood of getting another dose tain another dose. When quality and side effects were added as predictors, each had an independent effect on predicting continuation (Table 3). Having a higher quality initial provider interaction was significantly associated to the likelihood of getting another dose of DMPA-SC at 3 months (OR=2.039; 95% CI: 1.120–3.472). However, experiencing more bleeding reduced the likelihood of DMPA-SC continuation at 3 months (OR=0.154; 95% CI: 0.153–1.173).

**Table 2:** Sample characteristics and sociodemographic predictors of continued DMPA-SC use.

	N=311	**%**	Continued DMPA-SC	
			n/N=160/311 (49.7%)	
			OR	95% CI
Age				
<25	23	7.4%	Ref	
25–34	173	55.6%	1.554	(0.576–4.189)
35+	115	37.0%	1.188	(0.404–3.493)
Marital status				
Not currently married	9	2.9%	Ref	
Currently married	302	97.1%	3.239	(0.693–15.15)
Education				
Primary or less	42	13.5%	Ref	
Secondary	181	58.2%	1.433	(0.782–2.625)
College/University	88	28.3%	2.794**	(1.093–7.140)
Religion				
Muslim	97	31.2%	Ref	
Christian	214	68.8%	0.793	(0.470–1.339)
Parity				
0–1 children	43	13.8%	Ref	
2 children	75	24.1%	2.047	(0.866–4.838)
3 children	94	30.2%	2.476*	(0.953–6.436)
4+ children	99	31.8%	2.888**	(1.087–7.668)
Wealth quintile				
Poorest, poor, medium wealth	32	10.3%	Ref	
Wealthy	63	20.3%	1.237	(0.547–2.796)
Wealthiest	216	69.5%	1.134	(0.518–2.482)
Past contraception use				
New user	89	28.6%	0.807	(0.485–1.341)
Switched from a traditional method	47	15.1%	0.749	(0.391–1.432)
Switched from another injectable	102	32.8%	0.779	(0.438–1.386)
Switched from a non-injectable modern method	73	23.5%		
Place of purchase				
Private hospital/clinic/provider	43	13.8%	Ref	
Retail drug outlet	29	9.3%	1.471	(0.469–4.613)
DKT Bee	205	65.9%	0.976	(0.449–2.124)
Government hospital/clinic	34	10.9%	2.036	(0.746–5.557)
State				
All other states	220	70.7%	Ref	
Lagos	91	29.3%	0.764	(0.475–1.231)

Robust standard errors in parentheses, clustered by provider.

*** p<.01, ** p<.05, * p<.1.

**Table 3:** Logistic regressions of quality and side effects on continued DMPA-SC use.

	Continued DMPA-SC use
Quality	
Low quality	Ref
High quality	2.039***
	(1.120–3.472)
Side effects	
None	Ref
Less/no bleeding	0.708
	(0.381–1.318)
More bleeding	0.154***
	(0.069–0.)
Other	0.423*
	(0.153–1.173)
Observations	304

Robust standard errors in parentheses, clustered by provider.

*** p<.01, ** p<.05, * p<.1.

All regressions control for SES background characteristics.

## 4 Discussion

### 4.1 Main findings

Our findings indicate that higher quality of care at the time of initial counseling is associated with continuing DMPA-SC after 3 months. This suggests that, even in scenarios where women rate the overall quality of care as relatively high, small differences in the quality of care at the time of method uptake can influence contraceptive continuation. In this population, the lowest quality ratings were for items related to method choice: only a few women reported that their providers had little or no preference for which method she chose and about half were offered DMPA-SC as the only choice. The lack of method choice may be a result of providers being recently introduced to and/or trained on DMPA-SC, and thus more likely to promote it as a new product to the exclusion of other methods.

The strong association of the summary quality score with continued DMPA-SC use despite the limited variation in this overall quality measure also suggests that our conception of what constitutes “high quality” counseling in this setting may require further thought. This may be particularly true in terms of interpersonal dynamics, which were the highest-rated items within our quality measure. It is difficult to disentangle what type of “quality” the indictor for “how involved the provider was in decision making” is indicative of in the Nigerian setting. On the one hand, in a “Western” quality framework, researchers and practitioners may place high value on women's ability to make an informed decision and not be pressured by a provider [8]. Past studies in the United States exploring how involved women would like their provider to be in decision making about a method have found that women wanted the decision-making process to center around their preferences, although there were notable differences in how involved women wanted their provider to be by racial/ethnic group [44]. However, it is possible that in the Nigerian setting women may prefer to have the provider give more direction, particularly among lower-educated populations, in the absence of access to more information, and within sociocultural norms that defer decision-making to authoritative figures. Furthermore, one could argue that it is the provider's role to suggest a method that they think is most appropriate for a certain individual given their expertise. Indeed, many women in our sample stated that they highly valued the providers' recommendation as one of the main reasons why they chose to have the injection [36]. However, this assumes that providers are not unduly influenced by other incentives or the novelty and recent training on DMPA-SC to suggest one method over another.

Similarly, that the vast majority of the quality indicators had over 90% of women reporting “high” quality again suggests that these questions might not be appropriate for capturing heterogeneity in women's experiences of quality within this population. Past studies also found very positive responses to similar measures, which suggests that these measures may not be universally relevant or salient. Recent research in India and Kenya found that women value different aspects of quality of care for contraception than are often included in international measures. For example, waiting long periods of time was acceptable to some women in Kenya, while some women in India felt that having other patients in the room during exams was acceptable privacy [38]. Additional qualitative research is needed to further understand the meaning of quality to women in the Nigerian context.

Although over 70% of respondents indicated that the provider had asked or informed her about experiences with side effects, these aspects of information given were rated relatively lower in quality compared to other item responses and domains. Correspondingly, many women (~60%) reported side effects, especially irregular bleeding, as the main reason they discontinued DMPA-SC, which is consistent with other studies in Nigeria on the influence of side effects on continuing a variety of different methods, as well as studies of DMPA-related side effects and continuation [33], [34], [45]. However, it should be noted that women in our study were only surveyed after two injections spanning up to 6 months of protection against pregnancy; this short follow-up time frame may not have been sufficient for many women to experience side effects associated with long-term use of DMPA, such as amenorrhea [46]. Nonetheless, our results suggest that there is still much room for improvement in counseling quality, particularly on explaining side effects—whether experienced in the past or anticipated in the future—and that such improvements could have a large effect on DMPA-SC continuation.

### 4.2 Limitations

Our convenience sample of DMPA-SC users is unlikely to be representative of the Nigerian population of reproductive age women. In particular, due to lack of existing patient data from private sector providers and the challenges of systematically collecting such information, a representative sampling frame was not possible. Our sample was predominantly urban and only residing in the seven South West states; these women are likely to be wealthier and more educated than their national counterparts. As such, the examination of continuation is restricted to the subset of women who completed both surveys, who were more likely to be older and married compared to the full set of respondents to the baseline survey. While analyses of quality items between the follow-up cohort and those lost to follow-up did not show significant differences in responses across all item responses (see Supplementary materials), it should be recognized that younger, unmarried women nonetheless generally experience worse quality than older, married women [47]. Women were also only asked a small number of questions related to quality, which might not necessarily reflect their own perceptions of what constitutes quality for contraceptive services. Additional research is needed to define quality within specific populations. Participants' answers to the quality questions could be biased due to poor recall or social desirability, particularly if they suspected their responses to somehow be relayed back to the provider. It is also possible that providers only listed the contacts of certain types of clients that they thought would give good reports; however, we think this risk is low because providers did not know the nature of the survey for clients. While we trained interviewers to emphasize confidentiality for all providers and users, we cannot rule out these sources of potential bias. Finally, the current analysis only follows respondents for a short period after their initial dose and resource constraints prevented continued follow-up, even for women who stated that they intended to obtain a DMPA-SC reinjection at the time of the follow-up survey. Further analysis is needed to understand how quality is associated with continuation over longer time periods.

### 4.3 Conclusion

Contraceptive use remains low in Nigeria, and understanding how to increase method continuation is vital. While there is increasing focus on the effect of counseling quality on uptake and continuation, study results have been mixed. Our results support the notion that higher quality counseling can encourage women's continuation of a new injectable contraceptive method at 3 months. It also highlights the substantial role that side effects can have on women's decision to continue using a method. Future programs could focus on improving the quality of counseling about side effects, and particularly those related to bleeding (e.g., supporting women through their experience of side effects rather than relying on a brief mention during initial counseling) as this may help women expect and understand certain side effects and thus not be as likely to discontinue their method. New contraceptive methods designed for increased access and ease of use, combined with high quality provision, have potential for increased contraceptive use in settings with low contraceptive prevalence.

## Appendix A Supplementary data

Supplementary tables Image 1

## Footnotes

1 An additional five women did not respond to the question of how long ago they received their injection.

2 Because our sample is predominantly urban, we apply the urban asset weight index to yield a wealth distribution comparable to the Nigeria national distribution for urban areas per the Nigeria Demographic and Health Survey 2013 [35], following methods developed by Chakroborty et al. [43].
