# Supplementary material for: Predictors of DMPA-SC continuation among urban Nigerian women: the influence of counseling quality and side effects
Source: Contraception. 2018 Nov;98(5):430–7. doi: 10.1016/j.contraception.2018.04.015 (PMC6197834; doi:10.1016/j.contraception.2018.04.015)
Supplement: Supplementary file 1 — Supplementary tables [file mmc1.docx]

**Supplementary materials**

Table S1. Sociodemographic characteristics of the analytic sample compared to women excluded due to lost to follow-up

|  | Analytic sample (N=311) | | Excluded (N=190) | | p-value |
| --- | --- | --- | --- | --- | --- |
|  | N | **%** | N | **%** |  |
| Age |  |  |  |  |  |
| <25 | 23 | 7.4% | 31 | 16.3% | 0.000 |
| 25-34 | 173 | 55.6% | 101 | 53.2% |  |
| 35+ | 115 | 37.0% | 58 | 30.5% |  |
| Marital status |  |  |  |  |  |
| Not currently married | 9 | 2.9% | 8 | 4.2% | 0.000 |
| Currently married | 302 | 97.1% | 182 | 95.8% |  |
| Education |  |  |  |  |  |
| Primary or less | 42 | 13.5% | 31 | 16.3% | 0.759 |
| Secondary | 181 | 58.2% | 110 | 57.9% |  |
| College/University | 88 | 28.3% | 49 | 25.8% |  |
| Religion |  |  |  |  |  |
| Muslim | 97 | 31.2% | 61 | 32.1% | 0.839 |
| Christian | 214 | 68.8% | 129 | 67.9% |  |
| Parity |  |  |  |  |  |
| 0-1 children | 43 | 13.8% | 31 | 16.3% | 0.011 |
| 2 children | 75 | 24.1% | 47 | 24.7% |  |
| 3 children | 94 | 30.2% | 35 | 18.4% |  |
| 4+ children | 99 | 31.8% | 77 | 40.5% |  |
| Wealth quintile |  |  |  |  |  |
| Poorest, poor, medium wealth | 32 | 10.3% | 30 | 15.8% | 0.416 |
| Wealthy | 63 | 20.3% | 39 | 20.5% |  |
| Wealthiest | 216 | 69.5% | 121 | 63.7% |  |
| Past contraception use |  |  |  |  |  |
| New user | 89 | 28.6% | 54 | 28.6% | 0.257 |
| Switched from traditional method | 47 | 15.1% | 29 | 15.1% |  |
| Switched from another injectable | 102 | 32.8% | 62 | 32.8% |  |
| Switched from a non-injectable modern method | 73 | 23.5% | 45 | 23.5% |  |
| Place of purchase |  |  |  |  |  |
| Private hospital/clinic/provider | 43 | 13.8% | 22 | 11.6% | 0.657 |
| Retail drug outlet | 29 | 9.3% | 18 | 9.5% |  |
| DKT Bee | 205 | 65.9% | 127 | 66.8% |  |
| Government hospital/clinic | 34 | 10.9% | 23 | 12.1% |  |
| State |  |  |  |  |  |
| All other states | 220 | 70.7% | 10 | 5.3% | 0.478 |
| Lagos | 91 | 29.3% | 61 | 32.1% |  |

Table S2. Comparison of responses to quality measure items between the analytic sample and women excluded due to lost to follow-up

| Quality measure items | Analytic sample^1^ (N=311) | | Excluded from panel^1^  (N=229) | | p-value |
| --- | --- | --- | --- | --- | --- |
|  | N | % | N | % |  |
| **Provider asked if you had ever used contraception before** |  |  |  |  |  |
| No | 28 | 9 | 30 | 13.1 |  |
| Yes | 283 | 91 | 199 | 86.9 | 0.1291 |
| **Provider asked if you had ever experienced any side effects from another contraceptive** |  |  |  |  |  |
| No | 40 | 12.9 | 42 | 18.3 |  |
| Yes | 271 | 87.1 | 187 | 81.7 | 0.0798 |
| **Provider asked if you wanted to have more children in the future** |  |  |  |  |  |
| No | 33 | 10.6 | 35 | 15.4 |  |
| Yes | 278 | 89.4 | 192 | 84.6 | 0.0978 |
| **Provider asked if you had any health issue (e.g. infections, blood pressure)** |  |  |  |  |  |
| No | 74 | 23.8 | 62 | 27.4 |  |
| Yes | 237 | 76.2 | 164 | 72.6 | 0.3393 |
| **Provider described possible side effects of DMPA-SC** |  |  |  |  |  |
| No | 81 | 26 | 74 | 32.9 |  |
| Yes | 230 | 74 | 151 | 67.1 | 0.0849 |
| **Provider told you what to do if you have any problems with DMPA-SC** |  |  |  |  |  |
| No | 80 | 25.7 | 73 | 32.4 |  |
| Yes | 231 | 74.3 | 152 | 67.6 | 0.0894 |
| **Provider told you how long DMPA-SC protects against pregnancy** |  |  |  |  |  |
| No | 1 | 0.3 | 4 | 1.7 |  |
| Yes | 310 | 99.7 | 225 | 98.3 | 0.0878 |
| **Provider asked if you were pregnant/do anything to find out if you were pregnant** |  |  |  |  |  |
| No | 27 | 8.7 | 18 | 7.9 |  |
| Yes | 284 | 91.3 | 211 | 92.1 | 0.7335 |
| **Felt comfortable to ask questions** |  |  |  |  |  |
| No | 1 | 0.3 | 2 | 0.9 |  |
| Yes | 310 | 99.7 | 222 | 99.1 | 0.3836 |
| **Have enough privacy during the visit** |  |  |  |  |  |
| No | 11 | 3.5 | 12 | 5.2 |  |
| Yes | 300 | 96.5 | 217 | 94.8 | 0.3336 |
| **Felt that your waiting time was reasonable (vs. too long)** |  |  |  |  |  |
| Too long | 35 | 11.3 | 24 | 10.5 |  |
| No waiting time | 276 | 88.7 | 205 | 89.5 | 0.7763 |
| **Provider had no/slight preference for which method you should use (vs. moderate/strong)** |  |  |  |  |  |
| Moderate/Strong | 301 | 96.8 | 213 | 93.4 |  |
| No/Slight | 10 | 3.2 | 15 | 6.6 | 0.0668 |
| **Provider involved just right vs. too much/little** |  |  |  |  |  |
| Too much/too little | 7 | 2.3 | 7 | 3.1 |  |
| Just right | 304 | 97.7 | 221 | 96.9 | 0.5555 |
| **Provider mentioned more methods other than DMPA-SC** |  |  |  |  |  |
| Only DMPA-SC | 170 | 54.7 | 128 | 57.9 |  |
| More methods | 141 | 45.3 | 93 | 42.1 | 0.4568 |

^1^Samples are restricted to women who answered all quality measure question items.
